# Altered Effective Connectivity of Bilateral Hippocampus in Type 2 Diabetes Mellitus

**DOI:** 10.3389/fnins.2020.00657

**Published:** 2020-06-23

**Authors:** Taiyuan Liu, Yan Bai, Lun Ma, Xiaoyue Ma, Wei Wei, Junran Zhang, Neil Roberts, Meiyun Wang

**Affiliations:** ^1^Henan Key Laboratory of Neurological Imaging, Department of Medical Imaging, Zhengzhou University People’s Hospital & Henan Provincial People’s Hospital, Zhengzhou, China; ^2^Huaxi MR Research Center, Department of Radiology, West China Hospital of Sichuan University, Chengdu, China; ^3^The Queen’s Medical Research Institute, University of Edinburgh, Edinburgh, United Kingdom

**Keywords:** type 2 diabetes mellitus (T2DM), resting-state functional magnetic resonance imaging (rs-fMRI), Granger causality analysis (GCA), Effective Connectivity (EC), Hippocampus

## Abstract

Patients with type 2 diabetes mellitus (T2DM) experience cognitive deficits but the underlying pathophysiologic mechanisms are not known. We therefore applied Granger causality analysis of resting-state functional magnetic resonance imaging to study the effective connectivity (EC) of the hippocampus in patients with T2DM. Eighty six patients with T2DM and 84 matched healthy controls (HC) were recruited. The directional EC between anatomically defined seeds in left hippocampus (LHIP) and right hippocampus (RHIP) and other brain regions was compared between T2DM and HC and Pearson correlation analysis was performed to determine whether alterations in EC were related to clinical characteristics of diabetes. Compared with HC, patients with T2DM had altered EC between LHIP and RHIP and the default mode network (DMN), occipital cortex and cerebellum. In addition, for LHIP only duration of diabetes positively correlated with decreased inflow from right postcentral gyrus and right parietal lobe, glycosylated hemoglobin (HbA1c) negatively correlated with decreased inflow from right thalamus (*r* = −0.255, *p* = 0.018) and Montreal Cognitive Assessment (MoCA) negatively correlated with decreased inflow from left inferior parietal lobe (*r* = −0.206, *p* = 0.05). The altered EC between hippocampus and DMN is interpreted to be related to cognitive deficits in patients with T2DM particularly affecting memory and learning.

## Introduction

Diabetes is characterized by chronically increased peripheral insulin levels and concomitantly reduced brain insulin activity (Stolk et al., 1997; Baker et al., 2011). Type 2 diabetes mellitus (T2DM) is the most common form of diabetes, affecting approximately 400 million people worldwide (Chatterjee et al., 2017). Insulin resistance in T2DM disrupts many metabolic pathways, including glucose metabolism, synaptic maintenance, vascular function, tau phosphorylation, and β-amyloid regulation that support healthy cognitive functioning (Biessels et al., 1998; Gasparini et al., 2001; Schubert et al., 2004) and recent longitudinal studies have shown there is an associated increased risk of developing Alzheimer disease (AD) (Schrijvers et al., 2010; van Himbergen et al., 2012). The distribution of insulin receptors in the central nervous system is not uniform (Havrankova et al., 1978) and insulin resistance may compromise the function of specific brain regions including hippocampus, prefrontal cortex and cingulate gyrus. However, the underlying mechanisms have not been fully elucidated.

Multiple neuroimaging modalities such as functional magnetic resonance imaging (fMRI), single photon emission computed tomography (SPECT) and positron emission tomography (PET) have been used to study cognitive functions in patients with T2DM. Analysis of resting-state fMRI (rs-fMRI) data can be used to study the integration of specialized functional areas in the brain through measurement of functional connectivity (FC) and effective connectivity (EC). Measurement of FC reveals the functional connection between spatially distant brain regions, wheras EC measures the flow of information and the causal influence with Granger causality analysis (GCA) and provides the directionality and the dynamics of information dissemination within functionally related networks (Liao et al., 2010; Leavitt et al., 2012; Liao et al., 2019). Previous rs-fMRI studies in patients with T2DM have focused on measuring alterations in spontaneous neural activity (Cui et al., 2014) and the FC of posterior cingulate cortex (PCC; Chen et al., 2014) and hippocampus (Zhou et al., 2010). In some studies regions of the default mode network (DMN) such as PCC (Musen et al., 2012; Chen et al., 2014) and hippocampus (Zhou et al., 2010) have been used as seeds for FC analysis. However, EC studies of the directed influence of the hippocampus on other brain regions are likely to prove most effective for providing increased understanding of the physiopathologic mechanism in type 2 diabetes.

The hippocampus is known to mediate learning and different aspects of memory such as recognition memory and episodic-like memory (Morris, 2001; Fontan-Lozano et al., 2007). Previous MRI studies in patients with diabetes have provided evidence to suggest that impaired cognitive function is associated with alterations in hippocampal structure and function (Allen et al., 2007; Zhang et al., 2008). Furthermore, connectivity has been reported to be reduced between the hippocampus and other brain regions, including anterior cingulate cortex (ACC), medial temporal lobes and inferior parietal lobe (IPL), which are all part of the brain’s so-called default mode network (DMN) (Zhou et al., 2010). Since memory performance depends upon the function connectivity of multiple brain regions it has been suggested that hippocampal anomalies in T2DM may disrupt the ascending and descending pathways responsible for transmission and modulation of memory signals, and explain the impaired cognitive functions in T2DM.

The objective of the present study is to apply GCA of rs-fMRI data to compare the EC of the bilateral hippocampus and other brain regions in patients with T2DM relative to HC. Seed regions in the hippocampus are defined based on an anatomical atlas (Friston, 2009) and the hypothesis is tested that aberrant ascending and descending pathways at the hippocampus level may represent a physiopathologic mechanism underlying cognition deficits in T2DM.

## Materials and Methods

### Participants

This study was approved by the Research Ethics Committee (REC) of Henan Provincial People’s Hospital and written informed consent was provided by all participants. Eighty six patients with diabetes aged between 39 and 75 years and who were all right-handed were recruited together with 84 age, sex and handness-matched healthy controls (HC). The diagnosis of diabetes was made according to World Health Organization (WHO) 1999 criteria (American Diabetes Association, 2018). Subjects who had a history of alcoholism, smoking, stroke, brain injury, or other neurological or psychiatric disease that could lead to the cognition impairment, such as major depression, epilepsy, and Parkinson disease were excluded together with subjects who had severe hearing or visual impairment and any contraindication to MRI. The length of time for which patients had been diagnosed with diabetes, medication use, and medical history were recorded, together with height, weight and BMI ([weight in kg]/[height in meters]2), and arterial blood pressure was measured. Triglyceride (TR), total cholesterol, fasting plasma glucose (FPG), glycosylated hemoglobin (HbA1c), low density lipoprotein (LDL), and high density lipoprotein (HDL) were measured using blood samples collected at 7:00 am.

### Data Acquisition and Spatial Processing

MR data were acquired using a 3 T MRI system (Trio Tim, Siemens Healthineers, Erlangen, Germany) equipped with a standard 8-channel head coil. Head motion was minimized by the use of comfortable padding. For each subject a 3D T1-weighted image was acquired followed by rs-fMRI data using a gradient-echo planar imaging (EPI) sequence with voxel size 3.8 mm × 3.8 mm × 4.0 mm, TR 2,000 ms, TE 30 ms, matrix 64 × 64, Field of View (FOV) 240 mm × 240 mm, and slice thickness of 4 mm with no gap. The acquisition time was 7 min and 4 s. Subjects were instructed to rest quietly with their eyes closed and to avoid thinking about a specific subject.

Data analyses was performed using SPM8 (The Wellcome Department of Cognitive Neurology, London, United Kingdom)^1^. The first five time points of the rsfMRI data were discarded to ensure the signal had become stable and a slice timing correction was applied to the data for the remaining 195 time points. The images were subsequently realigned to correct for head motion and any images in which translation was >2 mm or rotation >2° were excluded. Following these pre-processing steps the mean image of the time series was spatially normalized into standard stereotactic space by registration to the Montreal Neurological Institute (MNI) EPI template and interpolated to isotropic voxels of 2 mm^3^ and smoothed using an isotropic Gaussian kernel with width of 4 mm. Next using data processing assistant for resting-state fMRI (DPARSF) software (Chao-Gan and Yu-Feng, 2010) a band-pass filter was applied (0.01 < f < 0.08 Hz) remove low frequency drift and high frequency noise. Further, linear regression of the average signals from white matter and cerebrospinal fluid (CSF) and six head motion parameters was applied to remove spurious variance from confounding factors not related to specific regional correlations (Fox et al., 2005). As a low model order was used in our GCA, the point that BOLD time series of regions of interest (ROIs) were not low-pass filtered should be noted.

### Granger Causality Analysis and Statistical Analysis

Also using DPARSF, GCA was applied to measure the EC between the time series of selected reference seed regions in left hippocampus (LHIP) and right hippocampus (RHIP), extracted using automatic anatomical labeling (AAL) atlas (Tzourio-Mazoyer et al., 2002) and the time series of every other voxel in the brain based on a hypothesis derived from previous studies which have reported bilateral alterations in the structure and function of the hippocampus in type 2 diabetes (Stankewitz et al., 2013; Zhao et al., 2013). In particular, the REST-GCA function in the REST toolbox of DPARSF was used to construct a bivariate first-order coefficient-based voxelwise GCA model (Song et al., 2011).

The GCA model is built based on the temporal elements of regional BOLD activity and describing the causal effect of the selected seed region (X) on every other voxel in the brain (Y) (i.e. X to Y effect). A positive coefficient indicates that activity in region X exerts a positive influence on activity in region Y (i.e. positive influence) whereas a negative coefficient indicates that the activity of region X exerts a negative influence on the activity of region Y. The voxel-wise GCA maps generated were normalized by transformation to z scores (Zang et al., 2012) and two-tailed, two-sample *t*-tests were performed to determine the significant differences in EC of LHIP and RHIP between patients with type 2 diabetes and HC. A Gaussian random field (GRF) correction was applied with voxel-level of *p* < 0.001 and joint cluster-level of *p* < 0.05. Age and sex ratio were included as covariates.

### Pearson Correlation Analysis and Statistical Analysis

The association between the altered EC of LHIP and RHIP and clinical variables measured for the patients with type 2 diabetes (disease duration, BMI, HbA1c, plasma glucose, and MoCA) were analyzed by using SPSS 17.0 (SPSS Inc., Chicago, IL, United States). In particular, ROIs corresponding to the brain regions which showing significantly different (i.e. increased or decreased) GCA influences between type 2 diabetes and HC were extracted and a Pearson correlation analysis was performed between mean GCA values within the ROIs and the clinical characteristics with threshold *p* ≤ 0.05.

## Results

### Demographic and Clinical Data

The demographic data obtained for all the participants and measures of clinical variables obtained for the patients with T2DM as diagnosed by two independent clinicians are presented in Table 1. Complete rs-fMRI datasets with no exclusions on account of, for example, head movement were obtained for all participants. No significant difference was found in the demographic data between the patients with type 2 diabetes and HC groups.

**TABLE 1:** Demographic characteristics and clinical measures.

	T2DM patients (*n* = 86)	Control subjects (*n* = 84)	P-value
Age (years)	54.08.9	54.88.5	0.52
Sex (male/female)	(44/42)	(42/42)	0.87
Height (cm)	167.30.07	166.00.07	0.25
Weight (kg)	71.810.9	70.010.2	0.30
BMI (kg/m^2^)	25.53.0	25.32.7	0.62
HbA1c (%)	8.11.7	–	–
Plasma glucose (mmol/L)	9.12.7	–	–
Diabetes duration(years)	8.76.1	–	–
Cholesterol (mmol/L)	4.41.0	4.40.7	0.79
Triglyceride (mmol/L)	2.12.1	1.81.1	0.27
Low density lipoprotein (mmol/L)	2.50.8	2.70.6	0.06
High density lipoprotein (mmol/L)	1.20.4	1.20.3	0.99
MoCA	25.02.0	25.42.0	0.19
Arterial blood pressure			
Systolic BP (mmHg)	120.66.6	119.55.2	0.23
Diastolic BP (mmHg)	78.55.2	78.14.7	0.59

*Data are means ± (SD).*

### Altered EC of LHIP

**FIGURE 1:**
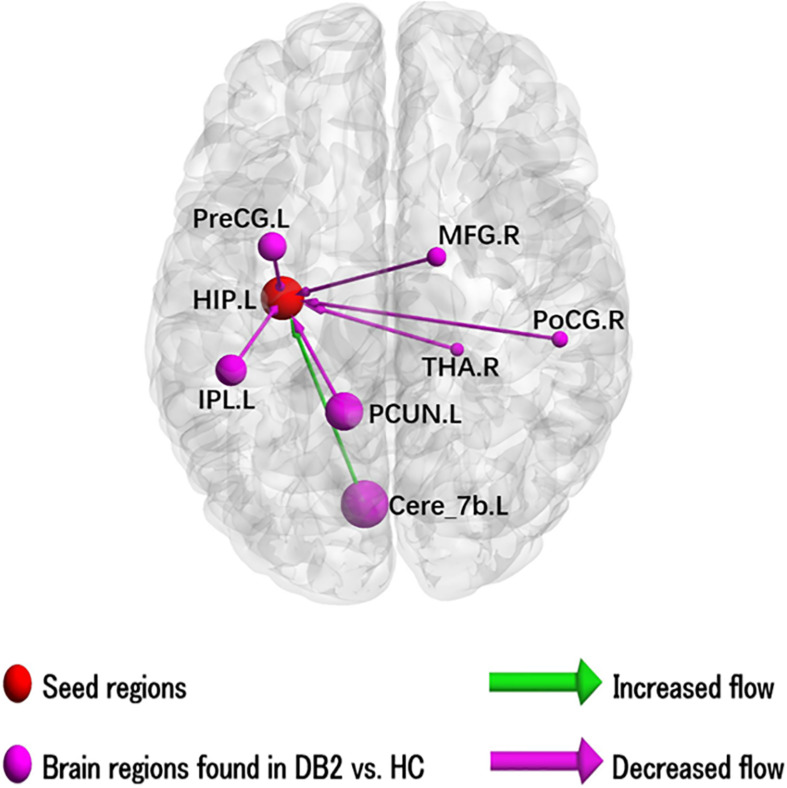
Illustration of the abnormal hippocampal Granger causality analysis model in type 2 diabetes compared with healthy control. Causal inflows are shown from the rest of the brain to left hippocampus. The node size is represented by number of voxels in the corresponding cluster in the rs-fMRI analysis. Cere, cerebellum; HIP, hippocampus; IPL, inferior parietal gyrus; L, left; MFG, middle frontal gyrus; PCUN, precuneus; PoCG, postcentral gyrus; PreCG, precentral gyrus; R, right; THA, thalamus.

The results of the two-sample *t*-test to compare EC of left HIP in patients with type 2 diabetes and HC are illustrated in Figure 1 and results for the ROI’s showing sigificant alterations in EC are listed in Supplementary Table 1. In patients with type 2 diabetes compared to HC, there is significantly increased causal inflow to LHIP from posterior lobe of left cerebellum (AAL: Cerebelum_7b_L) and significantly decreased causal inflow to LHIP from left precuneus (including the left parahippocampal, left fusiform), IPL and precentral gyrus and right thalamus, medial frontal gyrus (MFG), IPL and postcentral gyrus including supramarginal gyrus. There are no significant differences in outflows from LHIP.

### Altered EC of RHIP

**FIGURE 2:**
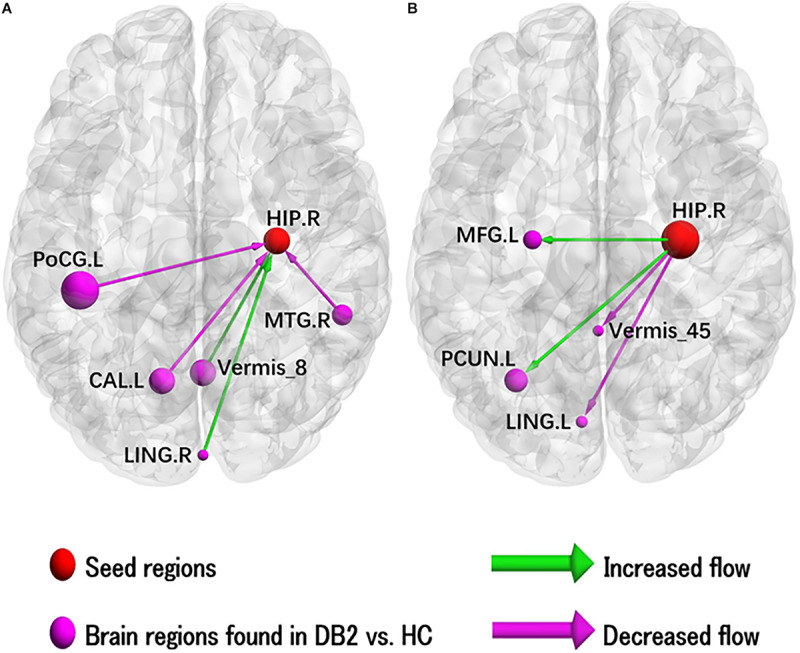
Illustration of the abnormal hippocampal Granger causality analysis model in type 2 diabetes compared with healthy control. **(A)** Causal inflows from rest of the brain to right hippocampus. **(B)** Causal outflows from right hippocampus to rest of brain. The node size is represented by number of voxels in the corresponding cluster in the rs-fMRI analysis. CAL, calcarine; HIP, hippocampus; L, left; LING, lingual; MFG, middle frontal gyrus; MTG, middle temporal gyrus; PCUN, precuneus; PoCG, postcentral gyrus; R, right.

The results of the two-sample *t*-test to compare EC of left HIP in patients with type 2 diabetes and HC are illustrated in Figure 2 and results for the ROI’s showing sigificant alterations in EC are listed in Supplementary Table 2. In patients with type 2 diabetes compared to HC, there is significantly increased causal inflow to RHIP from posterior lobe of right cerebellum and right lingual gyrus and significantly decreased causal inflow from left calcarine sulcus, post- and pre-central lobule and right MTG. In addition, patients with type 2 diabetes compared to HC also showed significantly increased outflow from RHIP to left precuneus, middle temporal lobe, middle occipital lobe, middle frontal gyrus, pre-central and post-central gyrus and significantly decreased outflow RHIP to anterior lobe of left cerebellum and left lingual gyrus.

### Correlation With Clinical Variables

The results of Pearson correlation analyses revealed that duration of diabetes was positively correlated with decreased inflow to LHIP from right postcentral gyrus (*r* = 0.225, *p* = 0.038) and right parietal lobe (*r* = 0.225, *p* = 0.039). In addition, there were significant negative correlations between HbA1c and decreased inflow to LHIP from right thalamus (*r* = −0.255, *p* = 0.018) and between MoCA and decreased inflow to LHIP from left parietal inferior (*r* = −0.206, *p* = 0.05). The other brain ROIs with altered EC between patients with type 2 diabetes and HC showed no significant correlation with the measurements of clinical variables (see Supplementary Figures 1–4).

## Discussion

To our knowledge this is the first study to apply GCA of rs-fMRI data and demonstrate alterations in EC of bilateral hippocampus in patients with T2DM compared to HC. Specifically, patients with T2DM had reduced/increased EC between LHIP and RHIP, and the DMN, occipital cortex and cerebellum. In addition, for LHIP only duration of diabetes positively correlated with decreased inflow from right postcentral gyrus and right parietal lobe, HbA1c negatively correlated with decreased inflow from right thalamus and MoCA negatively correlated with decreased inflow from left IPL.

The most prominent finding is perhaps the altered causal connectivity between bilateral hippocampus and the DMN, which most regions of DMN show decreased causal connectivity with bilateral hippocampus. The DMN is an anatomically separated but functionally connected network of brain structures and regions the activity of which is suspended when a subject performs a cognitive task. The core regions associated with the brain’s DMN mainly include ventral medial prefrontal cortex, posterior cingulate/retosplenial cortex, inferior parietal lobule, lateral temporal cortex, dorsal medial prefrontal cortex, and entorhinal cortex (Buckner et al., 2008). Parts of the DMN which have widespread functional connections with the hippocampus (Zhou et al., 2010) include ACC, PCC, MFG, MTG, precuneus, and IPL and which are thought to play a vital role in cognitive processing, such as episodic memory and executive function impairments (Buckner et al., 2008). Previous studies have shown that abnormalities in the structure and function of MTG within DMN may underlie cognitive impairment in T2DM (den Heijer et al., 2003; Chen et al., 2012; Xia et al., 2013), which is plausible since MTG is a critical node within DMN that supports verbal fluency, language processing and speech production (Pihlajamaki et al., 2000). medial frontal gyrus, another region of DMN, supports visual memory consolidation processes (Wang et al., 2008) and has been reported to have increased connectivity with the hippocampus in patients with AD in comparison to subjects with amnestic mild cognitive impairment (aMCI) and HC. This finding was interpreted as suggesting that a compensatory mechanism occurs in which increased FC of the MFG compensates for loss of FC in other regions of the DMN (Cha et al., 2013). Several brain regions were identified in the present study to possess increased connectivity with the hippocampus, suggesting perhaps that T2DM related cognitive impairment may be an early stage of AD-related cognitive impairment. Disruption of the DMN may be a potential mechanism whereby T2DM-related cognitive impairment develops gradually into AD.

Interestingly, the cerebellum was also found to show altered EC with the hippocampus bilaterally in patients with T2DM, and most of the causal connectivity was increased. There is convergent evidence to suggest that the cerebellum not only plays a vital role in motor function but is also involved in cognitive, emotional and sensory processing (Schmahmann et al., 2007; Stoodley and Schmahmann, 2009). Previous studies (Xia et al., 2013; Cui et al., 2014; Wang et al., 2014) reported inconsistent Amplitude of Low Frequency Fluctuation (ALFF) values in the cerebellum in T2DM. Xia et al. (2013) interpreted this as reflecting increased recruitment of additional neural resources in the cerebellum to compensate for loss of cognitive function in other regions. On the other hand, Bai et al. (2011) reported increased ALFF in the posterior lobe of the cerebellum lobe in a longitudinal study of subjects with aMCI patients. This is further indication that cognitive impairment in patients with T2DM may have a common mechanism to that in patients AD and which suggestion merits further study.

The findings of increased influence of the lingual gyrus and decreased influence of the calcarine sulcus on the HIP in patients with T2DM is consistent with a previous study in which it was reported that abnormal spontaneous brain acivity of lingual gyrus and calcarine sulcus were respectively closely correlated with impaired visual processing and visual spatial skills in T2DM patients (Peng et al., 2016). Wang et al. (2014) also reported finding abnormal ALFF values in brain regions associated with visual processing, particularly in the occipital lobe in patients with T2DM. Furthermore, in another study Degree Centrality (DC), a common measurement of the global connectivity of a network based on graph theory, was reported to be decreased in lingual gyrus and other occipital regions associated with visual function in T2DM (Cui et al., 2016). Retinopathy and neuropathy are common symptoms of diabetes that can cause visual and sensory impairment and it has been suggested that T2DM may cause alterations in the neural activity of the occipital lobe before the onset of visual impairment (Liu et al., 2011). Altered EC of lingual gyrus and calcarine sulcus may may be related to diabetic retinopathy and represent a potential neuropathic mechanism causing cognitive impairments in T2DM.

With regard to the analysis of potential correlation between altered hippocampal EC and clinical measures, for LHIP only duration of diabetes positively correlated with decreased inflow from right postcentral gyrus and right parietal lobe, which may be because of longer duration of T2DM leading to greater late-life cognitive decline. HbA1c negatively correlated with decreased inflow from right thalamus (*r* = −0.255, *p* = 0.018) and MoCA negatively correlated with decreased inflow from left IPL (*r* = −0.206, *p* = 0.05). The former finding may imply that improvement in glycemic control in T2DM is related to improved cognition. And the latter finding is potentially interpretable in that the IPL supports memory processing and thus decreased inflow from this brain region to the LHIP may contribute to poor memory performance of patients with T2DM.

There are several limitations that should be mentioned. Firstly, medications and other treatment that the patients with T2DM may have received could have had an impact on the findings that have been reported. Secondly, only the hippocampus was selected as a seed region for investigating pathways with altered EC. In future, the EC of more brain regions should be analyzed in order that a more comprehensive circuit model of cognitive impairmemt in T2DM may be constructed. Finally, the present study has a cross-sectional design and whether potential abnormalities of the pathways connecting hippocampus and the cerebral cortex are altered by disease duration and comorbidity remains to be determined.

In our study, the decreased causal connectivity of DMN and occipital regions with bilateral HIP may be related to cognitive deficits in patients with T2DM particularly affecting memory, learning and visual function, while the increasd causal connectivity of cerebellum mainly reflect the compensation for loss of cognitive function in other regions. In conclusion, GCA of rs-fMRI data has revealed significantly abnormal causal inflow and outflow to the bilateral HIP from various brain regions in patients with T2DM and which occur prior to alterations in the structure of these regions. The neural abnormalities were mainly located in the DMN, occipital cortex and cerebellum, and correlated with impairments in cognitive function. A new approach has been applied to investigate abnormalities in brain function in patients with T2DM and which provides enhanced understanding of the relationship between alterations in brain connectivity and cognitive impairment in patients with T2DM.

## Data Availability Statement

All datasets generated for this study are included in the article/Supplementary Material.

## Ethics Statement

The studies involving human participants were reviewed and approved by the Research Ethics Committee (REC) of Henan Provincial People’s Hospital. The patients/participants provided their written informed consent to participate in this study.

## Author Contributions

MW conceptualized and designed the study. TL was responsible for conducting the analyses, preparing a first draft of the manuscript, and preparing the manuscript for submission. LM, XM, and WW were responsible for data collection and initial data preprocessing. YB, NR, and JZ were responsible for data processing, supervising the analyses and editing drafts of the manuscript. All authors contributed to and approved the final manuscript.

## Conflict of Interest

The authors declare that the research was conducted in the absence of any commercial or financial relationships that could be construed as a potential conflict of interest.

## Footnotes

1 www.fil.ion.ucl.ac.uk/spm/software/spm8

## References

[B1] Allen G. Barnard H. McColl R. Hester A. L. Fields J. A. Weiner M. F. (2007). Reduced hippocampal functional connectivity in Alzheimer disease. *Arch. Neurol.* 64 1482–1487. 17923631 10.1001/archneur.64.10.1482

[B2] American Diabetes Association (2018). 2. Classification and Diagnosis of Diabetes: standards of Medical Care in Diabetes-2018. *Diabetes Care* 41(Suppl. 1), S13–S27. 29222373 10.2337/dc18-S002

[B3] Bai F. Liao W. Watson D. R. Shi Y. Yuan Y. Cohen A. D. (2011). Mapping the altered patterns of cerebellar resting-state function in longitudinal amnestic mild cognitive impairment patients. *J. Alzheimers Dis.* 23, 87–99. 10.3233/JAD-2010-101533 20930266

[B4] Baker L. D. Cross D. J. Minoshima S. Belongia D. Watson G. S. Craft S. (2011). Insulin resistance and Alzheimer-like reductions in regional cerebral glucose metabolism for cognitively normal adults with prediabetes or early type 2 diabetes. *Arch. Neurol.* 68 51–57. 20837822 10.1001/archneurol.2010.225 PMC3023149

[B5] Biessels G. J. Kamal A. Urban I. J. Spruijt B. M. Erkelens D. W. Gispen W. H. (1998). Water maze learning and hippocampal synaptic plasticity in streptozotocin-diabetic rats: effects of insulin treatment. *Brain Res.* 800 125–135. 10.1016/s0006-8993(98)00510-1 9685609

[B6] Buckner R. L. Andrews-Hanna J. R. Schacter D. L. (2008). The brain’s default network: anatomy, function, and relevance to disease. *Ann. N. Y. Acad. Sci.* 1112 1–38. 10.1196/annals.1440.011 18400922

[B7] Cha J. Jo H. J. Kim H. J. Seo S. W. Kim H. S. Yoon U. (2013). Functional alteration patterns of default mode networks: comparisons of normal aging, amnestic mild cognitive impairment and Alzheimer’s disease. *Eur. J. Neurosci.* 37 1916–1924. 10.1111/ejn.12177 23773060 PMC3694739

[B8] Chao-Gan Y. Yu-Feng Z. (2010). DPARSF: a MATLAB toolbox for “Pipeline” data analysis of resting-state fMRI. *Front. Syst. Neurosci.* 4:13. 10.3389/fnsys.2010.00013 20577591 PMC2889691

[B9] Chatterjee S. Khunti K. Davies M. J. (2017). Type 2 diabetes. *Lancet* 389 2239–2251. 28190580 10.1016/S0140-6736(17)30058-2

[B10] Chen Y. C. Jiao Y. Cui Y. Shang S. A. Ding J. Feng Y. (2014). Aberrant brain functional connectivity related to insulin resistance in type 2 diabetes: a resting-state fMRI study. *Diabetes Care* 37 1689–1696. 10.2337/dc13-2127 24658392

[B11] Chen Z. Li L. Sun J. Ma L. (2012). Mapping the brain in type II diabetes: voxel-based morphometry using DARTEL. *Eur. J. Radiol.* 81 1870–1876. 10.1016/j.ejrad.2011.04.025 21546180

[B12] Cui Y. Jiao Y. Chen Y. C. Wang K. Gao B. Wen S. (2014). Altered spontaneous brain activity in type 2 diabetes: a resting-state functional MRI study. *Diabetes Metab. Res. Rev.* 63 749–760. 10.2337/db13-0519 24353185

[B13] Cui Y. Li S. F. Gu H. Hu Y. Z. Liang X. Lu C. Q. (2016). Disrupted Brain Connectivity Patterns in Patients with Type 2 Diabetes. *AJNR Am. J. Neuroradiol.* 37 2115–2122. 10.3174/ajnr.a4858 27365332 PMC5201447

[B14] den Heijer T. Vermeer S. E. van Dijk E. J. Prins N. D. Koudstaal P. J. Hofman A. (2003). Type 2 diabetes and atrophy of medial temporal lobe structures on brain MRI. *Diabetologia* 46 1604–1610. 10.1007/s00125-003-1235-0 14595538

[B15] Fontan-Lozano A. Saez-Cassanelli J. L. Inda M. C. de los Santos-Arteaga M. Sierra-Dominguez S. A. Lopez-Lluch G. (2007). Caloric restriction increases learning consolidation and facilitates synaptic plasticity through mechanisms dependent on NR2B subunits of the NMDA receptor. *J. Neurosci.* 27 10185–10195. 10.1523/jneurosci.2757-07.2007 17881524 PMC6672666

[B16] Fox M. D. Snyder A. Z. Vincent J. L. Corbetta M. Van Essen D. C. Raichle M. E. (2005). The human brain is intrinsically organized into dynamic, anticorrelated functional networks. *Proc. Natl. Acad. Sci. U.S.A.* 102 9673–9678. 10.1073/pnas.0504136102 15976020 PMC1157105

[B17] Friston K. (2009). Causal modelling and brain connectivity in functional magnetic resonance imaging. *PLoS Biol.* 7:1000033. 10.1371/journal.pbio.1000033 19226186 PMC2642881

[B18] Gasparini L. Gouras G. K. Wang R. Gross R. S. Beal M. F. Greengard P. (2001). Stimulation of beta-amyloid precursor protein trafficking by insulin reduces intraneuronal beta-amyloid and requires mitogen-activated protein kinase signaling. *J. Neurosci.* 21 2561–2570. 10.1523/jneurosci.21-08-02561.2001 11306609 PMC6762523

[B19] Havrankova J. Roth J. Brownstein M. (1978). Insulin receptors are widely distributed in the central nervous system of the rat. *Nature* 272 827–829. 10.1038/272827a0 205798

[B20] Leavitt V. M. Wylie G. Genova H. M. Chiaravalloti N. D. DeLuca J. (2012). Altered effective connectivity during performance of an information processing speed task in multiple sclerosis. *Mult. Scler.* 18 409–417. 10.1177/1352458511423651 21965419

[B21] Liao W. Fan Y. S. Yang S. Li J. Duan X. Cui Q. (2019). Preservation Effect: cigarette Smoking Acts on the Dynamic of Influences Among Unifying Neuropsychiatric Triple Networks in Schizophrenia. *Schizophr. Bull.* 45 1242–1250. 10.1093/schbul/sby184 30561724 PMC6811814

[B22] Liao W. Mantini D. Zhang Z. Pan Z. Ding J. Gong Q. (2010). Evaluating the effective connectivity of resting state networks using conditional Granger causality. *Biol. Cybern.* 102 57–69. 10.1007/s00422-009-0350-5 19937337

[B23] Liu Y. Liang P. Duan Y. Jia X. Wang F. Yu C. (2011). Abnormal baseline brain activity in patients with neuromyelitis optica: a resting-state fMRI study. *Eur. J. Radiol.* 80 407–411. 10.1016/j.ejrad.2010.05.002 20605694

[B24] Morris R. G. (2001). Episodic-like memory in animals: psychological criteria, neural mechanisms and the value of episodic-like tasks to investigate animal models of neurodegenerative disease. *Philos. Trans. R. Soc. Lond. B Biol. Sci.* 356 1453–1465. 10.1098/rstb.2001.0945 11571036 PMC1088528

[B25] Musen G. Jacobson A. M. Bolo N. R. Simonson D. C. Shenton M. E. McCartney R. L. (2012). Resting-state brain functional connectivity is altered in type 2 diabetes. *Diabetes Metab. Res. Rev.* 61 2375–2379. 10.2337/db11-1669 22664957 PMC3425418

[B26] Peng J. Qu H. Luo T. Y. Lv F. J. Chen L. Wang Z. N. (2016). Abnormal spontaneous brain activity in type 2 diabetes with and without microangiopathy revealed by regional homogeneity. *Eur. J. Radiol.* 85 607–615. 10.1016/j.ejrad.2015.12.024 26860674

[B27] Pihlajamaki M. Tanila H. Hanninen T. Kononen M. Laakso M. Partanen K. (2000). Verbal fluency activates the left medial temporal lobe: a functional magnetic resonance imaging study. *Ann. Neurol.* 47 470–476. 10.1002/1531-8249(200004)47:4<470::aid-ana10>3.0.co;2-m 10762158

[B28] Schmahmann J. D. Weilburg J. B. Sherman J. C. (2007). The neuropsychiatry of the cerebellum - insights from the clinic. *Cerebellum* 6 254–267. 10.1080/14734220701490995 17786822

[B29] Schrijvers E. M. Witteman J. C. Sijbrands E. J. Hofman A. Koudstaal P. J. Breteler M. M. (2010). Insulin metabolism and the risk of Alzheimer disease: the Rotterdam study. *Neurology* 75 1982–1987. 10.1212/wnl.0b013e3181ffe4f6 21115952 PMC3014236

[B30] Schubert M. Gautam D. Surjo D. Ueki K. Baudler S. Schubert D. (2004). Role for neuronal insulin resistance in neurodegenerative diseases. *Proc. Natl. Acad. Sci. U.S.A.* 101 3100–3105. 14981233 10.1073/pnas.0308724101 PMC365750

[B31] Song X. W. Dong Z. Y. Long X. Y. Li S. F. Zuo X. N. Zhu C. Z. (2011). REST: a toolkit for resting-state functional magnetic resonance imaging data processing. *PLoS One* 6:e25031. 10.1371/journal.pone.0025031 21949842 PMC3176805

[B32] Stankewitz A. Schulz E. May A. (2013). Neuronal correlates of impaired habituation in response to repeated trigemino-nociceptive but not to olfactory input in migraineurs: an fMRI study. *Cephalalgia* 33 256–265. 10.1177/0333102412470215 23230239

[B33] Stolk R. P. Pols H. A. Lamberts S. W. de Jong P. T. Hofman A. Grobbee D. E. (1997). Diabetes mellitus, impaired glucose tolerance, and hyperinsulinemia in an elderly population. The Rotterdam Study. *Am. J. Epidemiol.* 145 24–32. 10.1093/oxfordjournals.aje.a009028 8982019

[B34] Stoodley C. J. Schmahmann J. D. (2009). Functional topography in the human cerebellum: a meta-analysis of neuroimaging studies. *Neuroimage* 44 489–501. 10.1016/j.neuroimage.2008.08.039 18835452

[B35] Tzourio-Mazoyer N. Landeau B. Papathanassiou D. Crivello F. Etard O. Delcroix N. (2002). Automated anatomical labeling of activations in SPM using a macroscopic anatomical parcellation of the MNI MRI single-subject brain. *Neuroimage* 15 273–289. 10.1006/nimg.2001.0978 11771995

[B36] van Himbergen T. M. Beiser A. S. Ai M. Seshadri S. Otokozawa S. Au R. (2012). Biomarkers for insulin resistance and inflammation and the risk for all-cause dementia and alzheimer disease: results from the Framingham Heart Study. *Arch. Neurol.* 69 594–600. 22213409 10.1001/archneurol.2011.670 PMC3512190

[B37] Wang C. X. Fu K. L. Liu H. J. Xing F. Zhang S. Y. (2014). Spontaneous brain activity in type 2 diabetics revealed by amplitude of low-frequency fluctuations and its association with diabetic vascular disease: a resting-state FMRI study. *PLoS One* 9:e108883. 10.1371/journal.pone.0108883 25272033 PMC4182760

[B38] Wang K. Jiang T. Yu C. Tian L. Li J. Liu Y. (2008). Spontaneous activity associated with primary visual cortex: a resting-state FMRI study. *Cereb. Cortex* 18 697–704. 10.1093/cercor/bhm105 17602140

[B39] Xia W. Wang S. Sun Z. Bai F. Zhou Y. Yang Y. (2013). Altered baseline brain activity in type 2 diabetes: a resting-state fMRI study. *Psychoneuroendocrinology* 38 2493–2501. 10.1016/j.psyneuen.2013.05.012 23786881

[B40] Zang Z. X. Yan C. G. Dong Z. Y. Huang J. Zang Y. F. (2012). Granger causality analysis implementation on MATLAB: a graphic user interface toolkit for fMRI data processing. *J. Neurosci. Methods* 203 418–426. 10.1016/j.jneumeth.2011.10.006 22020117

[B41] Zhang W. J. Tan Y. F. Yue J. T. Vranic M. Wojtowicz J. M. (2008). Impairment of hippocampal neurogenesis in streptozotocin-treated diabetic rats. *Acta Neurol. Scand.* 117 205–210. 10.1111/j.1600-0404.2007.00928.x 17854417

[B42] Zhao L. Liu J. Dong X. Peng Y. Yuan K. Wu F. (2013). Alterations in regional homogeneity assessed by fMRI in patients with migraine without aura stratified by disease duration. *J. Headache Pain* 14 1129–2377. 10.1186/1129-2377-14-85 PMC3853130 24134520

[B43] Zhou H. Lu W. Shi Y. Bai F. Chang J. Yuan Y. (2010). Impairments in cognition and resting-state connectivity of the hippocampus in elderly subjects with type 2 diabetes. *Neurosci. Lett.* 473 5–10. 10.1016/j.neulet.2009.12.057 20123114

