# Supplementary material for: Altered Effective Connectivity of Bilateral Hippocampus in Type 2 Diabetes Mellitus
Source: Front Neurosci. 2020 Jun 23;14:657. doi: 10.3389/fnins.2020.00657 (PMC7325692; doi:10.3389/fnins.2020.00657)
Supplement: Supplementary file 1 [file Data_Sheet_1.docx]

Supplementary Material

**1 Supplementary Figures**

**Supplementary Figure 1** GCA values show positive correlation with diabetes duration with decreased inflow from the right postcentral gyrus (in [51;-27;51] coordinate) to the left hippocampus (Pearson correlation, P≤0.05) in DB2 patients.

**Supplementary Figure 2** GCA values show positive correlation with diabetes duration with decreased inflow from the right parietal lobe (in [30;-51;39] coordinate) to the left hippocampus (Pearson correlation, P≤0.05) in DB2 patients.

**Supplementary Figure 3** GCA values show negative correlation in HbA1c values with decreased inflow from the right thalamus (in [21;-30;0] coordinate) to the left hippocampus (Pearson correlation, P≤0.05) in DB2 patients.

**Supplementary Figure 4** GCA values show negative correlation in MOCA values with decreased inflow from the left parietal inferior lobe (in [-45;-36;51] coordinate) to the left hippocampus (Pearson correlation, P≤0.05) in DB2 patients.

**2 Supplementary Tables**

**Supplementary** **Table 1** Two-sample t-test (voxel-level P < 0.05 and cluster-level P < 0.05 Gaussian random field corrected) of difference in causal influence to and from the left hippocampus in patients with DB2 versus HCs

| ***Regions*** | ***Peak voxel MNI coordinates(mm)*** | | | ***T values*** | ***Total***  ***voxels*** | ***Breakdown***  ***(number of voxels)*** |
| --- | --- | --- | --- | --- | --- | --- |
|  | ***x*** | ***y*** | ***z*** |  |  |  |
| **Causal inflow from the rest of the brain to left hippocampus(Y to X)** |  |  |  |  |  |  |
| Left Cerebellum // Cerebellum Posterior Lobe // Uvula //Cerebelum_7b_L | -6 | -75 | -42 | 3.9257 | 330 | Cerebelum_8_L (59)  Vermis_8 (44)  Cerebelum_Crus2_L (32)  Cerebelum_Crus2_R (28)  Cerebelum_Crus1_R (27)  Cerebelum_Crus1_L (25)  Cerebelum_6_L (24)  Calcarine_L (24)  Cerebelum_8_R (24)  Cerebelum_7b_L (18)  Cerebelum_9_R (14)  Lingual_R (11) |
| Left Cerebrum // Limbic Lobe // Posterior Cingulate // Precuneus_L | -12 | -48 | 9 | -4.1204 | 260 | Lingual_L (70)  Thalamus_L (31)  Precuneus_L (27)  Cerebelum_4_5_L (27)  Calcarine_L (27)  ParaHippocampal_L (25)  Fusiform_L (25)  Cuneus_L (15)  Hippocampus_L (13) |
| Right Cerebrum // Sub-lobar // Thalamus // Gray Matter | 21 | -30 | 0 | -4.4752 | 81 | Lingual_R (55)  Hippocampus_R (16)  ParaHippocampal_R (10) |
| Left Cerebrum // Parietal Lobe // Postcentral Gyrus // brodmann area 40 // Parietal_Inf_L | -45 | -36 | 51 | -3.6312 | 210 | Postcentral_L (85)  Parietal_Inf_L (49)  Temporal_Sup_L (23)  SupraMarginal_L (18)  Precentral_L (18)  Angular_L (17) |
| Right Cerebrum // Frontal Lobe // Medial Frontal Gyrus | 15 | -3 | 51 | -3.6948 | 123 | Cingulum_Mid_R (48)  Supp_Motor_Area_R (28)  Cingulum_Mid_L (25)  Cingulum_Ant_L (22) |
| Left Cerebrum // Frontal Lobe // Middle Frontal Gyrus // Precentral_L | -33 | 0 | 39 | -3.8544 | 192 | Frontal_Mid_L (91)  Precentral_L (85)  Frontal_Sup_L (16) |
| Right Cerebrum // Parietal Lobe // Sub-Gyral | 30 | -51 | 39 | -3.6467 | 61 | Postcentral_R (32)  SupraMarginal_R (15)  Parietal_Inf_R (14) |
| Right Cerebrum // Parietal Lobe // Postcentral Gyrus // Postcentral_R | 51 | -27 | 51 | -3.7266 | 101 | Postcentral_R (66)  Precentral_R (35) |

**Supplementary** **Table 2** Two-sample t-test (voxel-level P < 0.05 and cluster-level P < 0.05 Gaussian random field corrected) of difference in causal influence to and from the right hippocampus in patients with DB2 versus HCs

| ***Regions*** | ***Peak voxel MNI coordinates(mm)*** | | | ***T values*** | ***Total***  ***voxels*** | ***Breakdown***  ***(number of voxels)*** |
| --- | --- | --- | --- | --- | --- | --- |
|  | ***x*** | ***y*** | ***z*** |  |  |  |
| **Causal inflow from the rest of the brain to right hippocampus(Y to X)** |  |  |  |  |  |  |
| Right Cerebellum // Cerebellum Posterior Lobe // Uvula // Vermis_8 | 3 | -63 | -39 | 4.1642 | 291 | Cerebelum_Crus1_L (59)  Cerebelum_8_L (59)  Vermis_8 (43)  Cerebelum_6_L (42)  Cerebelum_7b_L (21)  Cerebelum_Crus2_L (21)  Cerebelum_9_R (18)  Cerebelum_8_R (16)  Cerebelum_10_L (12) |
| Right Cerebrum // Occipital Lobe // Lingual Gyrus | 3 | -93 | -9 | 3.679 | 41 | Cerebelum_Crus1_R (15)  Calcarine_L (15)  Cerebelum_Crus2_R (11) |
| Left Cerebrum // Limbic Lobe // Posterior Cingulate // brodmann area 30 // Calcarine_L | -12 | -66 | 9 | -4.2902 | 279 | Lingual_L (69)  Calcarine_L (67)  Thalamus_L (45)  Fusiform_L (34)  Cuneus_L (22)  ParaHippocampal_L (18)  Precuneus_L (12)  Hippocampus_L (12) |
| Right Cerebrum // Temporal Lobe // Superior Temporal Gyrus // Temporal_Mid_R | 54 | -42 | 9 | -3.9206 | 142 | Temporal_Mid_R (88)  Temporal_Sup_R (54) |
| Left Cerebrum // Parietal Lobe // Inferior Parietal Lobule // brodmann area 40 // Postcentral_L | -42 | -33 | 45 | -4.0294 | 1267 | Postcentral_L (180)  Precentral_L (178)  Frontal_Mid_L (141)  Parietal_Inf_L (121)  Temporal_Mid_L (115)  Parietal_Sup_L (77)  Occipital_Mid_L (74)  SupraMarginal_L (58)  Angular_L (57)  Cingulum_Mid_L (55)  Cingulum_Mid_R (51)  Temporal_Sup_L (40)  Supp_Motor_Area_L (40)  Insula_L (24)  Supp_Motor_Area_R (23)  Frontal_Sup_L (21)  Frontal_Inf_Oper_L (12) |
| Inter-Hemispheric // Corpus Callosum // Cingulum_Ant_L | 0 | 6 | 27 | -3.2929 | 29 | Cingulum_Ant_L (15)  Cingulum_Mid_R (14) |
| Right Cerebrum // Frontal Lobe // Inferior Frontal Gyrus // Frontal_Inf_Tri_R | 57 | 27 | 15 | -3.812 | 375 | Postcentral_R (183)  Precentral_R (101)  Frontal_Inf_Oper_R (31)  Frontal_Inf_Tri_R (29)  Parietal_Inf_R (21)  SupraMarginal_R (10) |
| **Causal inflow from right hippocampus to the rest of the brain (X to Y)** |  |  |  |  |  |  |
| Left Cerebellum // Cerebellum Anterior Lobe // Culmen // Vermis_4_5 | 0 | -48 | -12 | -3.4747 | 87 | Vermis_4_5 (27)  Cerebelum_6_R (17)  Vermis_3 (17)  Cerebelum_4_5_R (15)  Vermis_1_2 (11) |
| Left Cerebrum // Occipital Lobe // Lingual Gyrus // brodmann area 18 // Lingual_L | -6 | -81 | -12 | -3.7346 | 95 | Cerebelum_6_L (51)  Lingual_R (23)  Cerebelum_Crus1_L (11)  Lingual_L (10) |
| Left Cerebrum // Temporal Lobe // Sub-Gyral | -30 | -66 | 21 | 3.3788 | 184 | Precuneus_L (54)  Cingulum_Post_L (37)  Parietal_Sup_L (34)  Cuneus_L (21)  Temporal_Mid_L (20)  Occipital_Mid_L (18) |
| Left Cerebrum // Frontal Lobe // Sub-Gyral | -24 | -15 | 42 | 4.1804 | 148 | Frontal_Mid_L (61)  Precentral_L (47)  Postcentral_L (40) |
